# HCCANet: histopathological image grading of colorectal cancer using CNN based on multichannel fusion attention mechanism

**DOI:** 10.1038/s41598-022-18879-1

**Published:** 2022-09-06

**Authors:** Panyun Zhou, Yanzhen Cao, Min Li, Yuhua Ma, Chen Chen, Xiaojing Gan, Jianying Wu, Xiaoyi Lv, Cheng Chen

**Affiliations:** 1 College of Software, Xinjiang University, Urumqi, 830046 China; 2 The Affiliated Tumor Hospital of Xinjiang Medical University, Urumqi, 830011 China; 3 College of Information Science and Engineering, Xinjiang University, Urumqi, 830046 China; 4 Key Laboratory of Signal Detection and Processing, Xinjiang University, Urumqi, 830046 China; 5 Department of Oncology, Shanghai East Hospital, Tongji University School of Medicine, Shanghai, 200120 China; 6 Karamay Central Hospital of Xinjiang Karamay, Karamay, Xinjiang Uygur Autonomous Region, Department of Pathology, Karamay, 834000 China; 7 Xinjiang Cloud Computing Application Laboratory, Karamay, 834099 China; 8 College of Physics and Electronic Engineering, Xinjiang Normal University, Urumqi, 830054 China; 9 Key Laboratory of Software Engineering Technology, Xinjiang University, Urumqi, 830046 China

**Keywords:** Colorectal cancer, Image processing, Machine learning

## Abstract

Histopathological image analysis is the gold standard for pathologists to grade colorectal cancers of different differentiation types. However, the diagnosis by pathologists is highly subjective and prone to misdiagnosis. In this study, we constructed a new attention mechanism named MCCBAM based on channel attention mechanism and spatial attention mechanism, and developed a computer-aided diagnosis (CAD) method based on CNN and MCCBAM, called HCCANet. In this study, 630 histopathology images processed with Gaussian filtering denoising were included and gradient-weighted class activation map (Grad-CAM) was used to visualize regions of interest in HCCANet to improve its interpretability. The experimental results show that the proposed HCCANet model outperforms four advanced deep learning (ResNet50, MobileNetV2, Xception, and DenseNet121) and four classical machine learning (KNN, NB, RF, and SVM) techniques, achieved 90.2%, 85%, and 86.7% classification accuracy for colorectal cancers with high, medium, and low differentiation levels, respectively, with an overall accuracy of 87.3% and an average AUC value of 0.9.In addition, the MCCBAM constructed in this study outperforms several commonly used attention mechanisms SAM, SENet, SKNet, Non_Local, CBAM, and BAM on the backbone network. In conclusion, the HCCANet model proposed in this study is feasible for postoperative adjuvant diagnosis and grading of colorectal cancer.

## Introduction

Colorectal cancer (CRC), is a highly malignant tumor that forms in the tissues of the colon and rectum^1,2^. According to the American Cancer Society's Global Cancer Statistics 2021, there will be more than 1.9 million new cases of colorectal cancer and more than 900,000 deaths in 2020, making it the third leading cause of cancer death in the world, after lung cancer and breast cancer^3^. More than 90% of CRC cases are colorectal adenocarcinoma (CRA), which can be classified into grades I to IV according to Broder's criteria, i.e., highly differentiated (I), moderately differentiated (II), poorly differentiated (III), and undifferentiated (IV), based on the degree of glandular differentiation in histopathological images of colorectal cancer. The histological grading of colorectal cancer is not only a reference basis for assessing its malignancy and staging, but also an important factor affecting its prognosis^4^. Histopathological image analysis is the gold standard for pathologists to grade colorectal cancers of different differentiation types. In recent years, with the increasing number of colorectal cancer patients^1,2,5^, the workload of physicians is increasing day by day. In addition, the low differentiation of histopathological images of colorectal cancers of different differentiation types makes the diagnosis complicated and time-consuming^6^, which may lead to misdiagnosis and missed diagnosis^7^. Although gastroenterology clinics have a high demand for colon specimens, pathologists have a long training period (> 10 years)^8^. According to the Chinese Association of Pathologists, China, a country of 1.4 billion people, has only 20,000 professionally accredited pathologists^9^. Therefore, it is particularly important to build an efficient computerized automatic diagnostic model to effectively identify histopathological images of colorectal cancer at multiple levels, and then assist pathologist in objective diagnosis and grading.

With the rapid development of artificial intelligence technology in the medical field^10–12^ more and more CAD systems, especially convolutional neural network (CNN)-based CAD systems, are applied to automatic analysis tasks of histopathological images, such as cell nucleus detection and classification^13^, tumor segmentation^14^, tumor metastasis detection^15,16^, and cancer grading^17^. However, when faced with smaller medical image datasets, CNN models often fail to extract effective information from the dataset. This drawback makes it particularly important to combine CNN models with attention mechanisms^18^.

In this study, a new convolutional neural network and attention mechanism-based model, HCCANet, was proposed for grading colorectal cancers with different differentiation types. A total of 630 hematoxylin–eosin (H&E) stained histopathology images were included in this study, and the gaussian filtered images were fed into a fine-tuned VGG16 backbone network to extract local features. Then, the MCCBAM module is added in parallel to capture key features that facilitate network classification. Finally, the feature maps of the VGG16 and MCCBAM modules were fused to build the colorectal cancer supplementary diagnosis model HCCANet for the diagnosis of colorectal cancer of three grades: I, II, and III.

In general, the main contributions of this study can be summarized as follows:

1. This study constructs a new attention mechanism, called MCCBAM, based on multiple channel attention and spatial attention. This attention mechanism outperforms attention modules such as SAM, SENet, SKNet, Non_Local, CBAM, and BAM for classification on the fine-tuned VGG16 model.
2. In this study, a new automatic colorectal cancer diagnosis model based on a convolutional neural network and MCCBAM, called HCCANet, is proposed. This model enhances feature learning of key regions in histopathological images and outperforms advanced deep learning models and traditional machine learning algorithms in colorectal cancer grading tasks.
3. In this study, the Grad-CAM visualization method was introduced to convert the model output into a heat map, visualize key regions of interest for the model, enhance the interpretability of the model, and assist pathologists in investigating misdiagnosis cases of false negative and false positive.

## Related work

CNN models automatically learn features from input images and build low-level features into high-level features, and have had great success in computer vision fields such as image classification, image segmentation, and object detection tasks^19–21^. Recently, an increasing number of researchers have used CNNs as an aid in the diagnosis of colorectal cancer. Yoon et al. proposed an improved VGG model for classifying normal and tumor tissue from 10,280 colorectal histological images with an accuracy of 82%^22^. Ponzio et al. used a pre-trained VGG16 model for migration learning to classify colorectal histopathology images into normal, adenoma, and adenocarcinoma categories and obtained 96% classification accuracy^23^. Nguyen et al. used a combined model of classical CNN and CapsNet to classify histopathological images of 410 patients into three categories: tumor, normal epithelium, and other tissue types, achieving a multi-classification accuracy of 95.3%^24^. Zhou et al. proposed a new cell-graph convolutional neural network (CGC-Net) to classify colorectal histopathological images into low-grade cancer (Highly differentiated and moderately differentiated colorectal cancer) and high-grade cancer (Poorly differentiated and undifferentiated colorectal cancer), obtaining 91.60% accuracy on patch images^25^. Shaban et al. proposed a new context-aware neural network for grading colorectal pathological tissue images (normal, low-grade cancer, high-grade cancer) and obtained an average accuracy of 95.70%^26^. The above-mentioned studies on colorectal cancer grading have classified colorectal cancer into two grades: high-grade cancer and low-grade cancer^25,26^, but in the actual treatment process, some studies need to classify colorectal cancer into four grades: I (highly differentiated), II (moderately differentiated), III (undifferentiated), and IV (undifferentiated)^4^. In addition, some deep learning models based on histopathological images do not perform well on small medical datasets, or even as well as traditional machine learning^27,28^.

Attention mechanisms (AM) derived from human intuition have been widely used in computer vision, and AM allocates computational resources to the most informative parts of the signal, bringing significant improvements to many visual processing tasks. For example, tasks such as image classification^29^, object detection^30^, action recognition^31^, pose estimation^32^, and super-resolution^33^. At present, some investigators have introduced the attention mechanism into the CAD system of colorectal cancer. Pei et al. proposed a model based on a convolutional neural network and attention mechanism to automate colorectal cancer tumor segmentation, which includes a channel attention module and a location attention module to obtain more contextual information in the deeper layers of the network^34^. Chen et al. proposed a weakly supervised colorectal histopathology image classification model based on interactive learning and multichannel attention mechanisms, which identifies attention regions as accurately as possible in both channel and spatial dimensions by integrating different attention mechanisms^35^. Although the attentional mechanisms used in the above studies performed well for tasks such as segmentation and classification, they performed poorly for histological grading of colorectal cancer.

In summary, a new attention mechanism called MCCBAM was constructed in this study, and a new model based on convolutional neural network and MCCBAM, HCCANet, was proposed to assist in the diagnosis of histopathological images of colorectal cancer with three different differentiation types: high, medium and low.

## Materials and methods

### Materials

**Figure 1:**
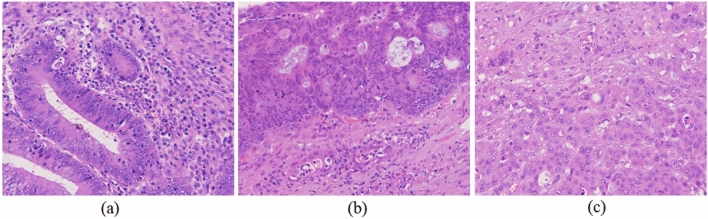
Different differentiation types of colorectal cancer by digital pathological imager at 40 times magnification. (**a**) Highly differentiated. (**b**) Moderately differentiated. (**c**) Highly differentiated.

In this study, 105 patients were enrolled in the Cancer Hospital of Xinjiang Medical University between 2012 and 2021, including 35 patients each with colorectal cancer of grades I, II, and III differentiation. The grading of colorectal cancer is based on the degree of glandular differentiation, with grades I, II, and III corresponding to > 95%, 50–95%, and 5–50% of glandular differentiation, respectively. The histological sections of colorectal cancers included in the study were confirmed by postoperative tissue biopsy and were retrieved from the pathology department of the hospital, and two experienced histopathologists labeled the ROI of each patient's tissue section. Sixty of these patients were male (age range, [36–85]) and 45 were female (age range, [23–71]). Histopathological images of colorectal cancers are shown in Fig. 1.

The acquisition of the final image used in the experiment consists of two steps. First, the pathological tissue sections were placed horizontally under the digital pathology imager, and two experienced histopathologists selected and labeled ROIs on each patient's tissue section in turn. Second, six non-overlapping images of 1665 × 1393 pixels were extracted from the ROIs at 40 × magnification, and 210 images of each of the three differentiation grades of colorectal cancer, high, medium, and low, were extracted, totaling 630 images. The child images extracted from the histopathological images of the parent images were verified by the pathologist to be of the same differentiation level as the parent image. The details of patients are shown in Table 1.

**Table 1:** Patient information sheet.

Information	Value
**Gender**	
Male	60 (number)
Female	45 (number)
**Age**	
Male	57.97 (average age)
Female	55.23 (average age)
**Number of patients**	
High differentiation (I)	35
Medium differentiation (II)	35
Low differentiation (III)	35
**Number of images**	
High differentiation	210
Medium differentiation	210
Low differentiation	210

### Data augmentation

Due to the limited number of samples in the dataset used in this study, a deep neural network trained with a dataset of this size is risky, and the network is likely to be overfitted due to the small dataset. Therefore, the number of training images is increased using data augmentation methods. First, the data set is divided into a training set, validation set, and test set according to the ratio of 8:1:1. Second, the training set is augmented to 4500 sheets, including rotation, cropping, scaling, etc.

### Image processing for model training

Image pre-processing work can improve the performance of the model to some extent^36^. In this study, the image pre-processing work includes three points, first, the image noise processing: this study uses four filtering techniques: mean filtering, median filtering, gaussian filtering, and bilateral filtering to de-noise the image respectively. The best filtering technique was selected by comparing the performance of the images on HCCANet after different filtering techniques. Second, image resizing: the original pixel size of 1665 × 1393 is scaled to 224 × 224 to better fit the backbone network in the HCCANet model^7,37,38^. Third, image normalization: The image is normalized by calling the image preprocessing method *scale()* of the Sklearn.preprocessing module. The *scale()* method subtracts the data by its attributes from its mean value and divides it by its variance so that all data for each attribute are clustered around 0 and the variance value is 1.

### Methods

**Figure 2:**
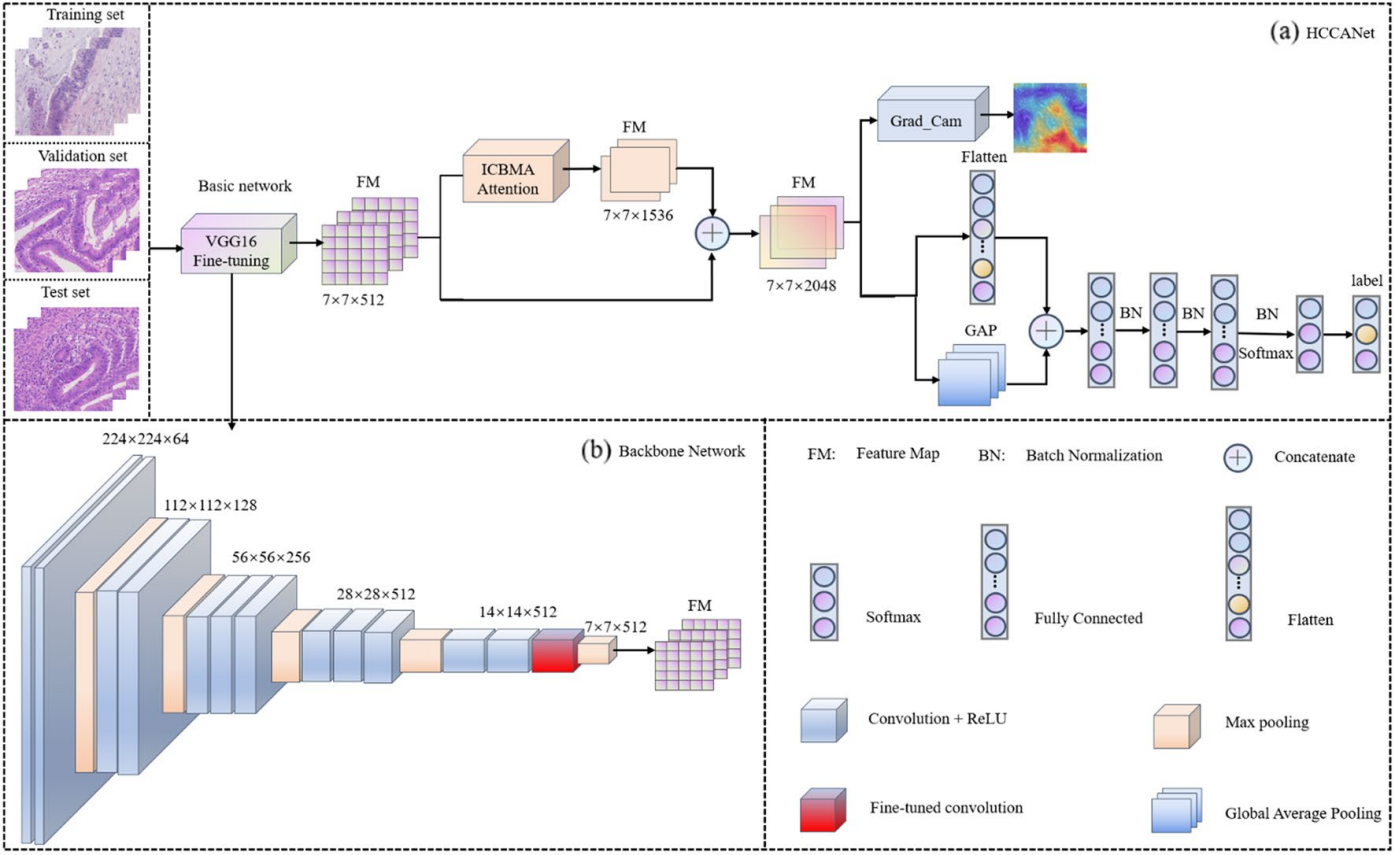
The framework of HCCANet. (**a**) The overall architecture of HCCANet. (**b**) The backbone of HCCANet.

In this study, a cleverly designed network structure named HCCANet is proposed to perform the task of grading histopathological images of colorectal cancer of different differentiation types. Figure 2a shows the overall architecture of HCCANet. Figure 2b shows the visual attention mechanism module MCCBAM constructed in this paper.

HCCANet consists of two parts, the backbone network VGG16 and the multichannel converged attention mechanism MCCBAM. In the medical field, one of the main challenges in adopting deep learning models is the lack of training data due to the difficulty in collecting and labeling data^39^. To make the VGG16 model more applicable to the dataset in this study and reduce the risk of overfitting the model on small datasets, we migrate the weights of the VGG16 model trained on the ImageNet dataset and fine-tune the *Block_Conv3* layer of the VGG16 model (the module marked in red in Fig. 2b).

### MCCBAM attention mechanism

**Figure 3:**
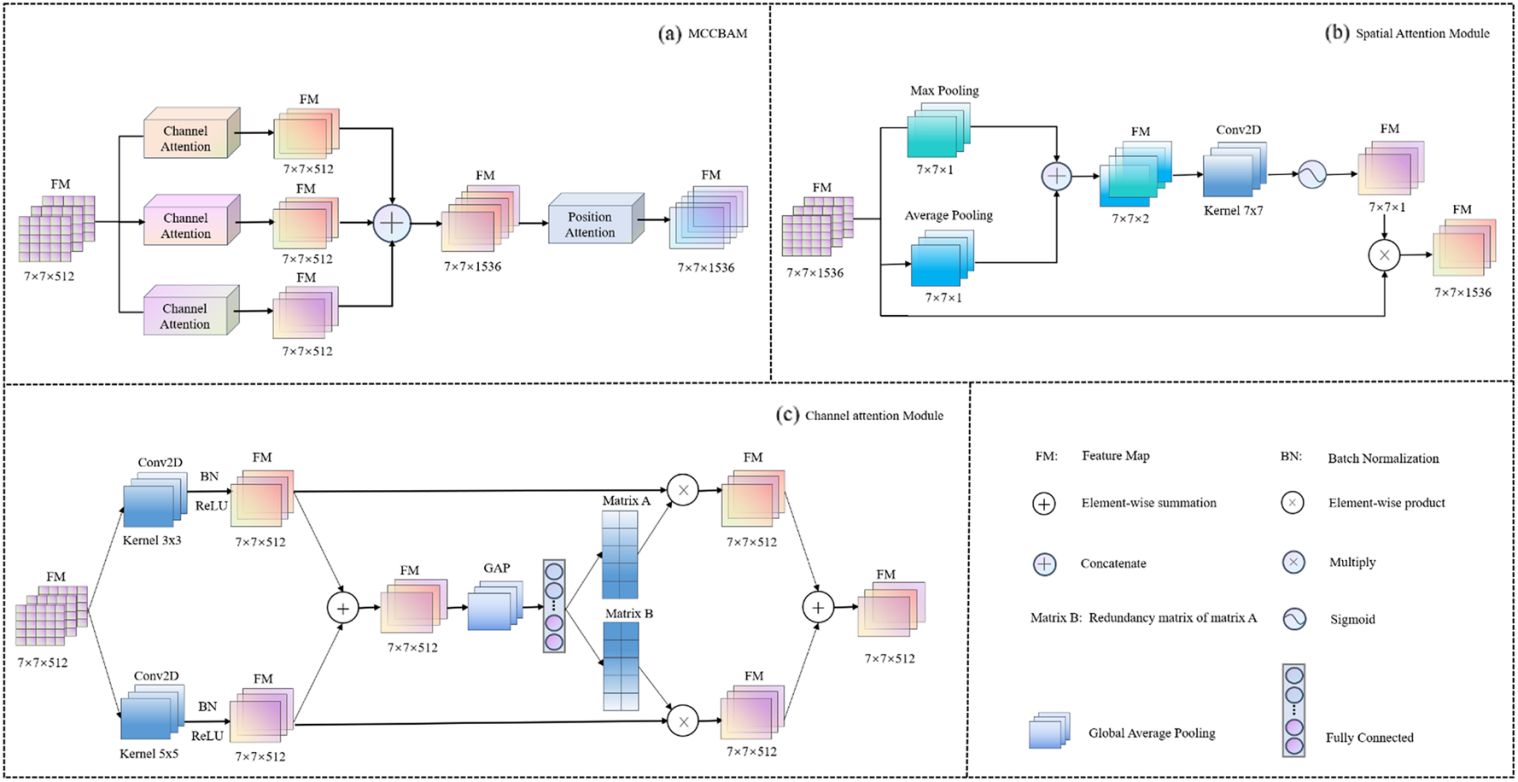
The framework of MCCBAM. (**a**) The overall architecture of MCCBAM. (**b**) Components of the Spatial Attention Block. (**c**) Components of the Channel Attention Block.

AM has become one of the most essential concepts in the field of deep learning^40^. However, traditional AMs have some drawbacks. For example, individual AMs may have difficulty in capturing useful features, AMs may capture redundant information, etc. To reduce the drawbacks of AMs, we construct a new attention module called MCCBAM. The module consists of three parallel SKNets^41^ and Spatial Attention Mechanism (SAM)^29^, and Fig. 3a shows the overall architecture of MCCBAM.

The MCCBAM module consists of two parts, namely SKNet and SAM. The processing of features in the MCCBAM module consists of two steps: First, the features are processed by SKNet with three reduction ratios of 4, 8, and 16, and the processed features are fused by concatenating. Second, the fused features are fed into the spatial attention mechanism with a kernel size of 7 for further processing. As shown in Fig. 3a, given an intermediate feature map $FM\in {R}^{H * w * C}$ as input, MCCBAM sequentially derives three-channel attention maps $F{M}_{c}\in {R}^{H * W * c}$ and one spatial attention map $F{M}_{s}\in {R}^{H * W * 1}$, and the whole attention process can be summarized as follows:

1 $$F\left(FM\right)={FM}_{\mathrm{s }}\left([{ {FM}_{c}^{r = 4}\left(FM\right);{FM}_{c}^{r = 8}\left(FM\right);{FM}_{c}^{r = 16}\left(FM\right)}] \otimes FM\right)\otimes FM$$

where $F(FM) \in {R}^{H * W * 3c}$ is the final output feature map, the superscript r in $F{M}_{c}^{r}$ represents the magnitude of the reduction ratio, and $\otimes$ means the element multiplication.

The essence of the channel attention module comes from the squeeze and excitation network^42^. Its essence is to allow the network to use global information to selectively enhance beneficial feature channels and suppress useless ones, thus enabling adaptive channel selection. SKNet is a new channel attention mechanism in which each neuron in the module adapts the size of its receptive field to capture target objects at different scales according to the multiple scales of the input information^41^. As shown in Fig. 3c, given an intermediate feature map $FM\in {R}^{H * w * C}$ as input, channel attention derives a channel attention map $F{M}_{c}\in {R}^{H * W * c}$, and the whole attention process can be summarized as:

2 $${M}_{\mathrm{a }}= FC\left(GAP\left({f}^{3 * 3}\left(FM\right) \oplus { f}^{5 * 5}\left(FM\right) \right)\right), { M}_{\mathrm{b}}=1- {M}_{\mathrm{a}}$$

3 $${FM}_{\mathrm{c }}\left(FM\right)=\left({M}_{\mathrm{a }} \otimes FM \right) \oplus \left({M}_{\mathrm{b }} \otimes FM\right)$$

where $GAP$ denotes Global Average Pooling, $FC$ denotes Fully Connected, ⊕ means element summing, ${M}_{\mathrm{a}}$ and ${M}_{\mathrm{b}}$ represent matrices.

Unlike channel attention, spatial attention adopts a global perspective to learn the connections between voxels and tasks, focusing on the spatial location information of key features by establishing rich contextual relationships between local features and assigning different weights to them^43^. As shown in Fig. 3b, given an intermediate feature map $FM\in {R}^{H * w * C}$ as input, the spatial attention is derived as a spatial attention map $F{M}_{s}\in {R}^{H * W * 1}$, and the whole attention process can be summarized as:

4 $${FM}_{\mathrm{s }}(\mathrm{FM})=\sigma \left({f}^{7 * 7}\left([{ AvgPool\left(FM\right); MaxPool\left(FM\right)}]\right)\right)=\sigma \left({f}^{7 * 7}\left([{F}_{avg}^{s}; {F}_{max}^{s}]\right)\right)$$

where $\sigma$ denotes sigmoid function, ${f}^{7 * 7}$ represents a convolution operation with the filter size of 7 × 7, $AvgPool$ and $MaxPool$ represent the average pooling and maximum pooling operations, respectively.

### CNN-based classifiers for comparison

This study used four advanced deep learning models ResNet50, MobileNetV2, Xception, and DenseNet121 to build the classifier. Inspired by Tajbakhsh^44^, four CNN models were fine-tuned using weights pre-trained on ImageNet. Araújo^27^ and Yan^28^ showed that features extracted with a pre-trained CNN were able to achieve better performance than some end-to-end CNN classifiers on SVM classifiers. Therefore, this study uses a pre-trained VGG16 network to extract features and uses the extracted features to train classifiers such as KNN, RF, NB, and SVM. The classification performance of the above models is compared with that of HCCANet, and the specific experimental configuration is shown in Table 2.

**Table 2:** Hyperparameter settings for each classifier.

Model name	Hyper-parameters
**Deep learning algorithms**	
ResNet50	Input size: (224, 224, 3), Learning rate: 0.005, Epochs: 100 Optimizer: Adam (${\beta }_{1}{=0.9, \beta }_{2}=0.99$ 9), Batch size: 32 Loss function: Categorical Cross-Entropy
MobileNetV2	
Xception	
DenseNet121	
**Traditional machine learning algorithms**	
KNN	Neighbors: 5
RF	Estimators: 850, Random state: 0, Bootstrap: True
NB	Alpha: 1.0
SVM	Kernel: RBF, C: 1.0, Gamma: 0.005

### Performance evaluation

In this study, receiver operating characteristic (ROC) curves and confusion matrices were plotted to assess the performance of HCCANet in terms of accuracy and reliability. Area under curve (AUC) is a quantitative measure of the model's performance, and the closer the value of AUC is to 1, the better the model performs. In addition, we also calculate the precision, recall, F1-score, and accuracy of the model when predicting samples to evaluate the model, and these metrics are calculated as shown in Table 3.

**Table 3:** Performance metric calculation formulas.

Performance metric	Precision	Recall	F1-score	Accuracy
Formula	$\frac{\mathrm{TP}}{\mathrm{TP}+\mathrm{FP}}$	$\frac{\mathrm{TP}}{\mathrm{TP}+\mathrm{FP}}$	$2*\frac{\mathrm{Precision}*\mathrm{Recall}}{\mathrm{Precision}+\mathrm{Recall}}$	$\frac{\mathrm{TP}+\mathrm{TN}}{\mathrm{TP}+\mathrm{TN}+\mathrm{FP}+\mathrm{FN}}$

### Informed consent

This study has been approved by the Cancer Affiliated Hospital of Xinjiang Medical University (in these studies). Informed consent was obtained from all participants before participating in the interview study. All methods were carried out in accordance with relevant guidelines and regulations (e.g. Helsinki guidelines). This article is based on the project "PI3K/AKT and MEK/ERK signaling pathways in microRNA-106b-induced epithelial transformation process in colorectal cancer cells the process of mesothelial transformation in colorectal cancer cells", which was approved by the ethics committee of the Cancer Hospital of Xinjiang Medical University, so the article does not require a separate ethics report.

## Results

All experiments in this study are based on the Python programming language, using TensorFlow-GPU deep learning framework to build the deep learning models needed during the experiments, and using GeForce RTX 1080ti for training. The Sklearn machine learning library was used to build the machine learners needed for the experiments. All classifiers were trained using a five-fold cross-validation method.

### Selection of filters or image denoising

**Figure 4:**
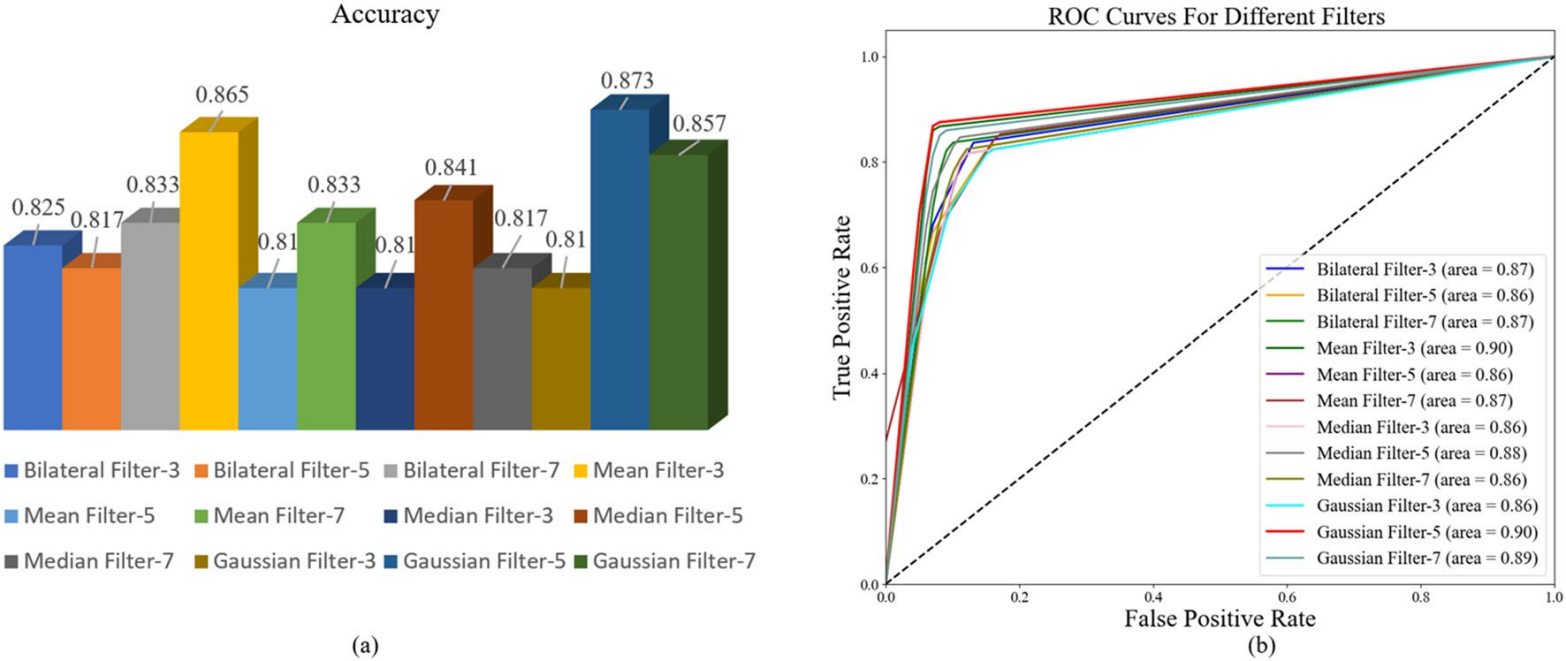
(**a**) Accuracy of HCCANet based on different filters. (**b**) AUC values for HCCANet based on different filters.

Medical images usually have a noise component, and the removal of this noise is essential for medical diagnosis^45^. To reduce the impact of the noise present on medical images on the classification performance of the model, four filtering techniques, namely, mean filter, median filter, Gaussian filter, and bilateral filter, are used in this study to de-noise the images respectively. A cross-sectional comparison of the precision, recall, F1-score, and accuracy of HCCANet based on different filtering techniques on histopathological images of colorectal cancer with different differentiation types was performed to select the optimal filter. From Table 4, it can be seen that choosing a Gaussian filter with a kernel size of 5 can improve the ability of HCCANet for automatic diagnosis of colorectal cancer histopathology images. Figure 4a,b shows the performance of HCCANet based on different filters in terms of accuracy and AUC values, respectively. (See Supplementary Material for the confusion matrix).

**Table 4:** Comparison of the denoising effect of different filtering techniques.

Filter type/kernel size	Grading	Precision	Recall	F1-score	Accuracy
Mean filtering/3	I	0.87	0.952	0.91	0.865
	II	0.872	0.81	0.84	
	III	0.854	0.833	0.843	
Mean filtering/5	I	0.868	0.786	0.825	0.810
	II	0.775	0.738	0.756	
	III	0.792	0.905	0.844	
Mean filtering/7	I	1.00	0.810	0.895	0.833
	II	0.816	0.738	0.775	
	III	0.741	0.952	0.833	
Median filtering/3	I	0.804	0.881	0.841	0.810
	II	0.767	0.786	0.776	
	III	0.865	0.762	0.810	
Median filtering/5	I	0.860	0.881	0.871	0.841
	II	0.795	0.833	0.814	
	III	0.872	0.810	0.840	
Median filtering/7	I	0.826	0.905	0.864	0.817
	II	0.861	0.738	0.795	
	III	0.773	0.810	0.791	
Bilateral filtering/3	I	0.860	0.881	0.871	0.825
	II	0.848	0.667	0.747	
	III	0.780	0.929	0.848	
Bilateral Filtering / 5	I	0.854	0.833	0.843	0.817
	II	0.729	0.833	0.778	
	III	0.892	0.786	0.835	
Gaussian filtering/3	I	0.917	0.786	0.846	0.810
	II	0.723	0.810	0.764	
	III	0.814	0.833	0.824	
Gaussian filtering/5	I	0.902	0.881	0.892	0.873
	II	0.850	0.810	0.829	
	III	0.867	0.929	0.897	
Gaussian filtering/7	I	0.860	0.881	0.871	0.857
	II	0.833	0.833	0.833	
	III	0.878	0.857	0.867

### Comparison of MCCBAM with other attention mechanisms

**Figure 5:**
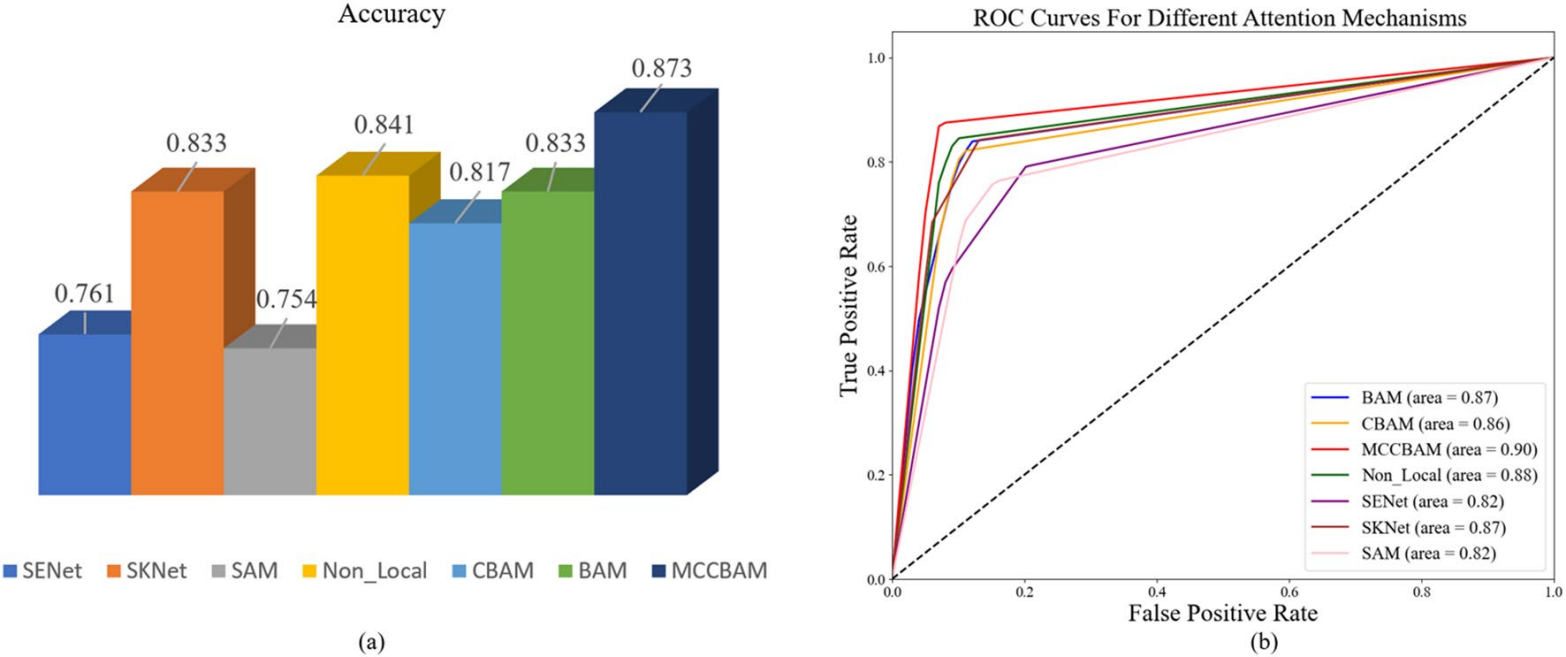
(**a**) Accuracy of VGG16 based on different attention mechanisms. (**b**) AUC values of VGG16 based on different attention mechanisms.

To evaluate the performance of the MCCBAM attention mechanism constructed in this study on the histopathological image grading of colorectal carcinoma, we incorporated six commonly used attention mechanisms, SAM, SENet^42^, SKNet, Non_Local^31^, CBAM^29^, and BAM to construct a comparison experiment. All attention mechanisms are added to the tail of the same backbone network in a parallel manner. The experimental results showed that the model based on the MCCBAM attention mechanism outperformed other attention mechanisms in terms of precision, recall, F1-score, and accuracy, demonstrating the superiority and usability of the MCCBAM attention mechanism constructed in this study for histopathological image grading of colorectal carcinoma. The experimental results are shown in Table 5. Figure 5a,b shows the performance of the VGG16 backbone network based on different attention mechanisms in terms of accuracy and AUC values, respectively. (See Supplementary Material for the confusion matrix).

**Table 5:** Comparison of MCCBAM with other attention mechanisms.

Attention mechanism	Grading	Precision	Recall	F1-score	Accuracy
SAM	I	0.769	0.714	0.741	0.754
	II	0.698	0.714	0.706	
	III	0.795	0.833	0.814	
SENet	I	0.696	0.929	0.796	0.762
	II	0.806	0.595	0.685	
	III	0.821	0.762	0.790	
SKNet	I	0.878	0.857	0.867	0.833
	II	0.761	0.833	0.795	
	III	0.872	0.810	0.840	
Non_Local	I	0.857	0.857	0.857	0.841
	II	0.846	0.786	0.815	
	III	0,0.822	0.881	0.851	
CBAM	I	0.850	0.810	0.829	0.817
	II	0.786	0.786	0.786	
	III	0.818	0.857	0.837	
BAM	I	0.923	0.857	0.889	0.833
	II	0.767	0.786	0.776	
	III	0.818	0.857	0.837	
MCCBAM	I	0.902	0.881	0.892	0.873
	II	0.850	0.810	0.829	
	III	0.867	0.929	0.897

### CNN-based classifiers for comparison

**Figure 6:**
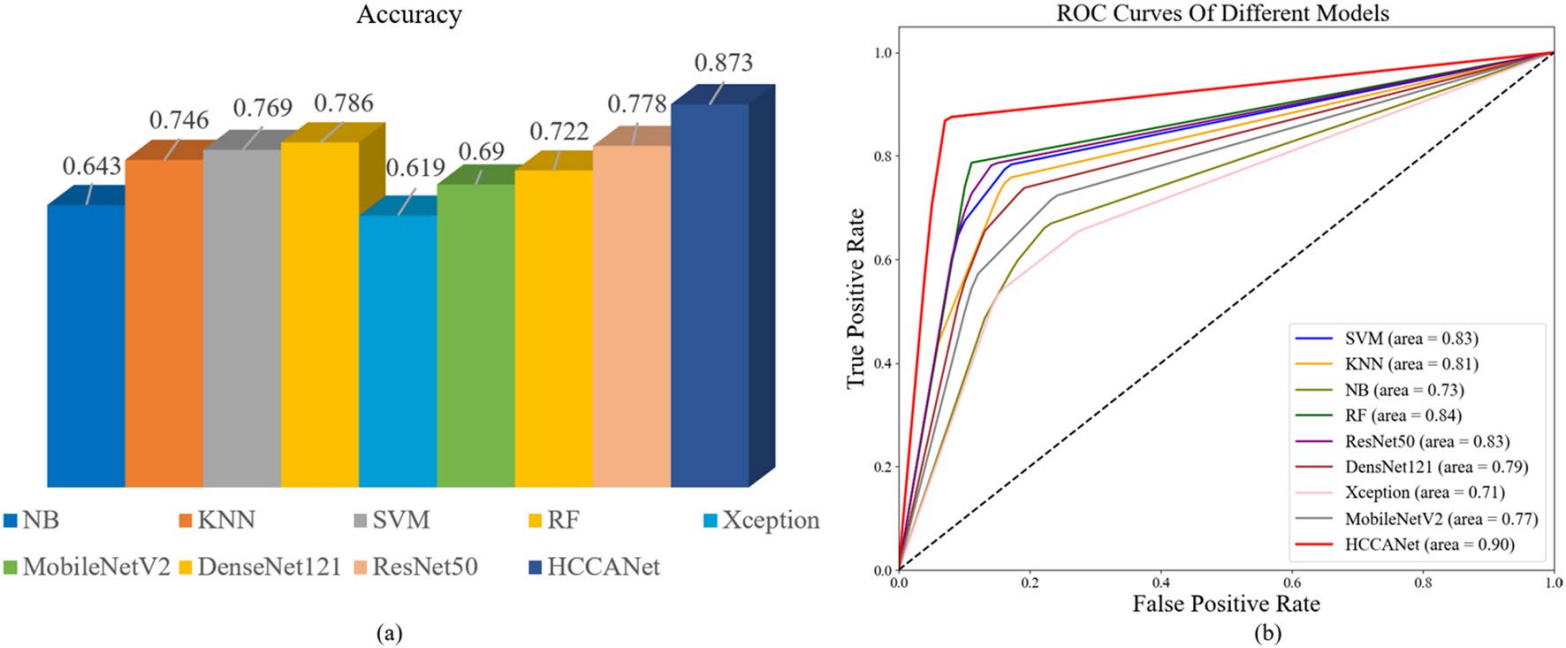
(**a**) Accuracy of different models in grading histopathological images. (**b**) AUC values for different models on histopathological image grading.

This study uses four advanced deep learning models ResNet50, MobileNetV2, Xception, and DenseNet121, and four commonly used machine learning models KNN, RF, NB, and SVM to build classifiers for training and compare them with HCCANet. From Table 6, the average classification accuracy of HCCANet is 9.5% higher than that of ResNet50, 18.3% higher than that of MobileNetV2, 25.4% higher than that of Xception, 15.1% higher than that of DenseNet121, 12.7% higher than that of KNN, 8.7% higher than that of RF, 23% higher than that of NB, and 10.4% higher than that of SVM 10.4%. The experimental results show that HCCANet outperforms other classifiers in terms of precision, recall, F1-score, and accuracy, proving the superiority and usability of the HCCANet model for histopathological image grading of colorectal cancer. Figure 6a,b shows the accuracy and AUC values of different models for grading histopathological images, respectively. (See Supplementary Material for the confusion matrix).

**Table 6:** CNN-based classifiers for comparison.

Classifier	Grading	Precision	Recall	F1-Score	Accuracy
ResNet50	I	0.786	0.786	0.786	0.778
	II	0.714	0.714	0.714	
	III	0.833	0.833	0.833	
MobileNetV2	I	0.727	0.571	0.640	0.690
	II	0.608	0.738	0.667	
	III	0.762	0.762	0.762	
Xception	I	0.676	0.595	0.632	0.619
	II	0.511	0.571	0.539	
	III	0.690	0.690	0.690	
DenseNet121	I	0.789	0.714	0.750	0.722
	II	0.644	0.690	0.667	
	III	0.744	0.762	0.753	
KNN	I	0.865	0.762	0.810	0.746
	II	0.675	0.643	0.659	
	III	0.714	0.833	0.769	
RF	I	0.791	0.810	0.800	0.786
	II	0.757	0.667	0.709	
	III	0.804	0.881	0.841	
NB	I	0.703	0.619	0.658	0.643
	II	0.583	0.500	0.583	
	III	0.642	0.810	0.716	
SVM	I	0.805	0.786	0.795	0.769
	II	0.689	0.738	0.713	
	III	0.825	0.786	0.805	
HCCANet	I	0.902	0.881	0.892	0.873
	II	0.850	0.810	0.829	
	III	0.867	0.929	0.897

### Grad-CAM visual analysis

**Figure 7:**
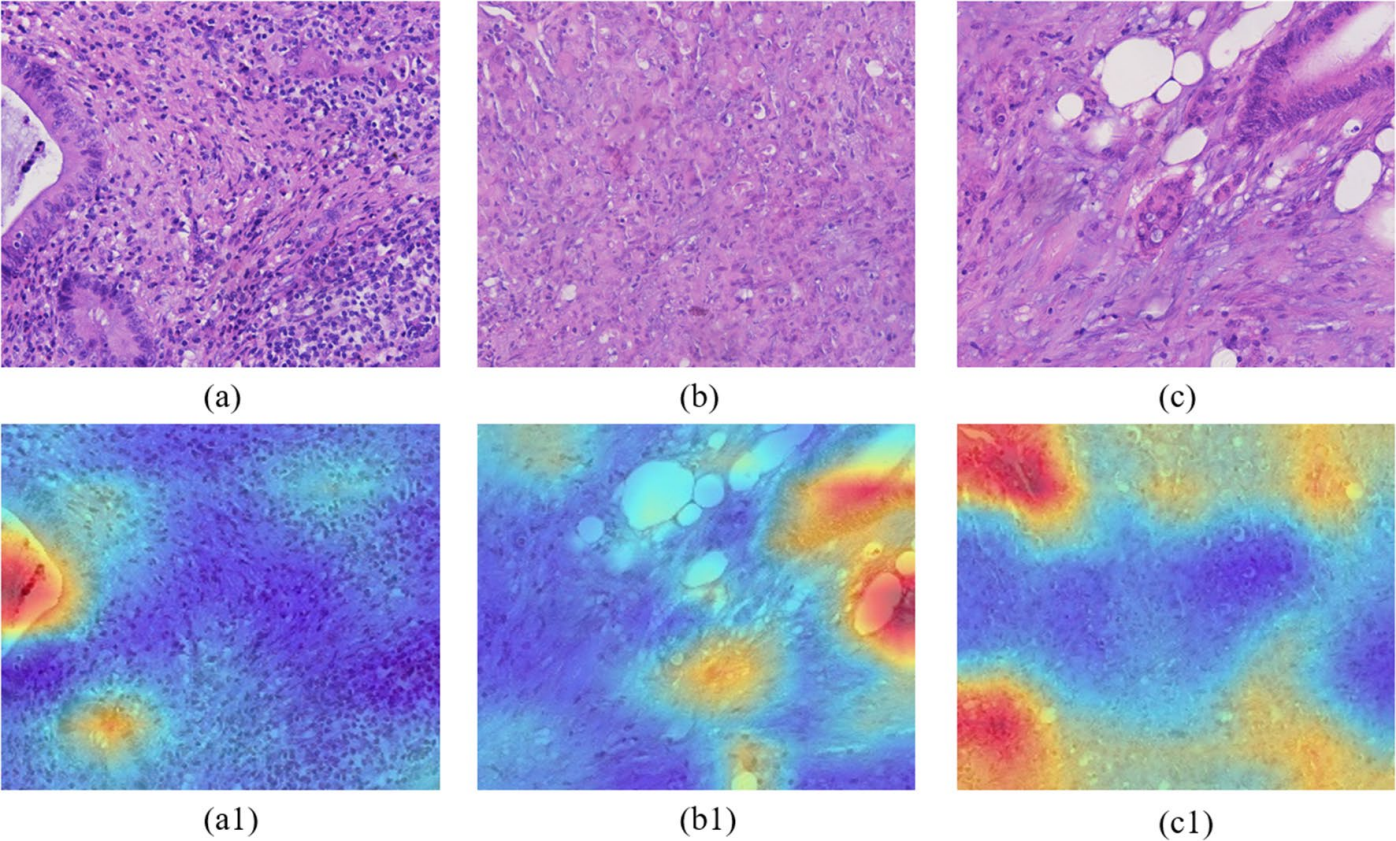
Histopathological images of colorectal cancer and its corresponding CAMs.

Grad_CAM is a widely used method for visualizing feature maps that uses gradients to calculate the importance of spatial locations in a convolutional layer^46^. In the heat map generated by Grad_CAM, the blue color is the unimportant region, while the red color is the critical region associated with that category. The classifier makes judgments based on these local pixel-level features in red. As shown in Fig. 7, the upper part is the histopathological image of colorectal cancer stained by H&E, and the lower part is the Grad-CAMs generated by HCCANet after extracting relevant features. Figure 7a–c shows the images of colorectal cancer at grade I, grade II, and grade III stages (i.e., highly differentiated, moderately differentiated, and poorly differentiated stages, respectively), Fig. 7(a1), (b1) and (c1) are their corresponding Grad-CAMs.

## Discussion

In this study, we propose a new computer-aided diagnostic model that can use histopathological images to distinguish three differentiation grades of colorectal cancer: high, intermediate, and low. Due to the scarce and precious nature of medical images, the model often fails to perform as expected in solving actual disease diagnosis using advanced deep learning models. To address these issues, we use image denoising, image enhancement, weight migration and model fine-tuning to make the deep learning models perform better on the dataset in this study. In addition, this study constructs a new attention mechanism, called MCCBAM, based on channel attention and spatial attention, which outperforms multiple current state-of-the-art attention mechanisms in the colorectal cancer 3-level grading task in this study, resulting in a large improvement in the discriminative power of the model.

This study combines MCCBAM and a fine-tuned VGG16 network architecture to construct a new model for histological grading of colorectal cancer, called HCCANet. To our knowledge, this is the first study that combines deep learning and attentional mechanisms to grade colorectal cancer of different differentiation types. The model has a good performance on the dataset used in this study, and the classification performance is better than existing deep learning models and classical machine learning models, which has some practical value in solving the realistic problems of manual grading of colorectal cancer after surgery to some extent. In addition, the gradient-weighted class activation map (Grad-CAM) visualization method is used to display the fused feature maps, which can improve the interpretability of the model and better help pathologists understand the output feature maps of HCCANet.

In the future, we will continue to collect more samples from different center institutions to build a companion diagnostic model with better performance and higher generalization ability, so as to make greater use of the clinical significance of postoperative diagnosis of colorectal cancer. In addition, we plan to combine histopathological images with clinical data to build a complementary diagnostic model based on multimodal information.

## Supplementary Information

Supplementary Information.

## Data Availability

The datasets generated and analyzed during the current study are not publicly available due to data privacy laws, but are available from the corresponding author on reasonable request.
