# Supplementary material for: HCCANet: histopathological image grading of colorectal cancer using CNN based on multichannel fusion attention mechanism
Source: Sci Rep. 2022 Sep 6;12:15103. doi: 10.1038/s41598-022-18879-1 (PMC9448811; doi:10.1038/s41598-022-18879-1)
Supplement: Supplementary file 1 — Supplementary Information. [file 41598_2022_18879_MOESM1_ESM.pdf]

Supplementary material for different classifier confusion matrices

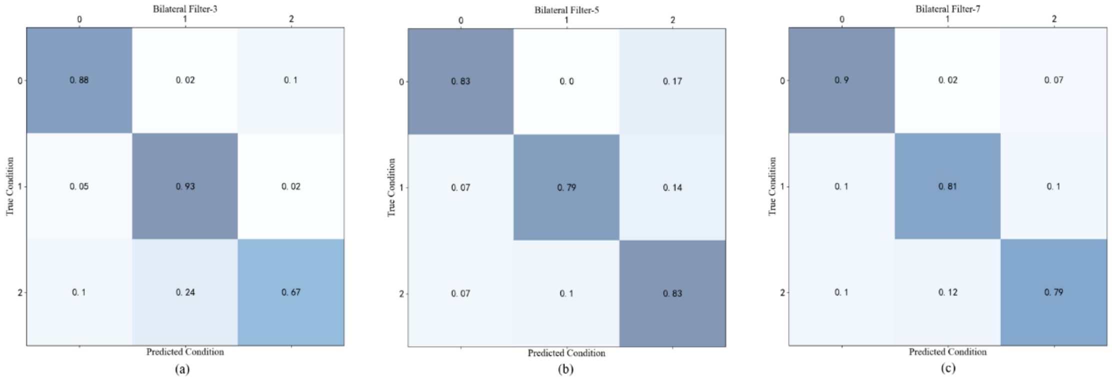

Fig.S1 (a), (b), (c) represent bilateral filters with kernel sizes of 3, 5 and 7 respectively

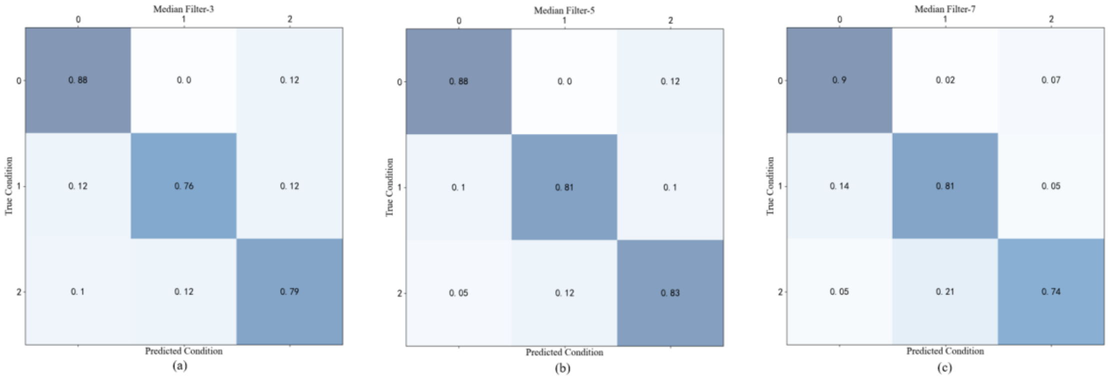

Fig. S2 (a), (b), (c) represent median filters with kernel sizes of 3, 5 and 7 respectively

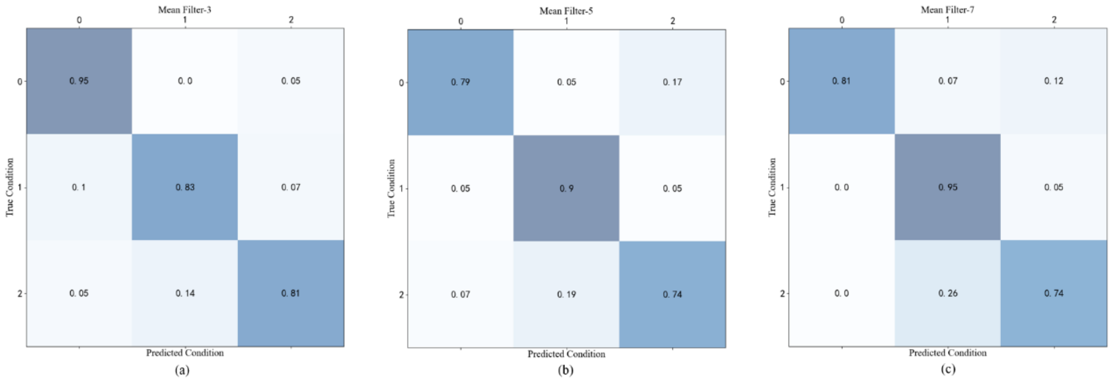

Fig. S3 (a), (b), (c) represent mean filters with kernel sizes of 3, 5 and 7 respectively

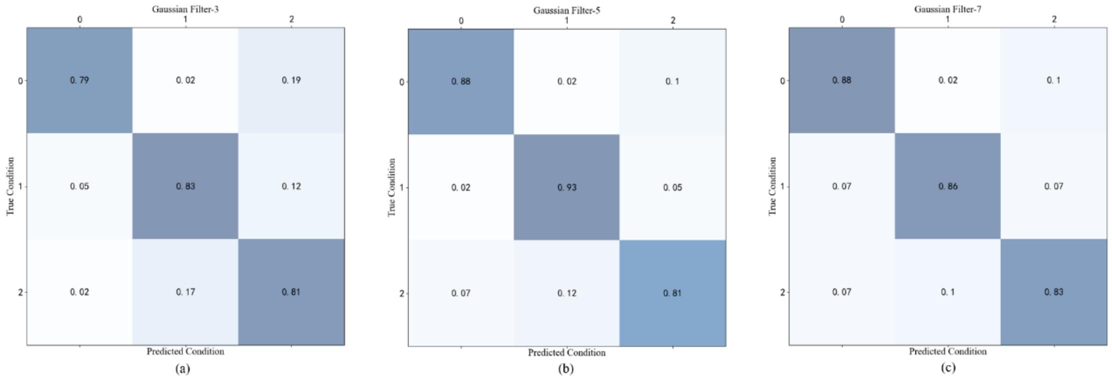

Fig. S4 (a), (b), (c) represent gaussian filters with kernel sizes of 3, 5 and 7 respectively

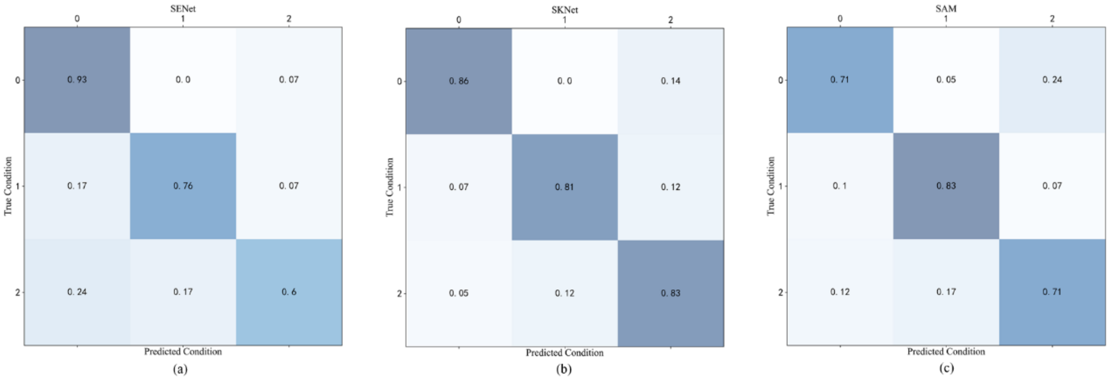

Fig. S5 (a), (b), (c) represent SENet, SKNet, SAM respectively

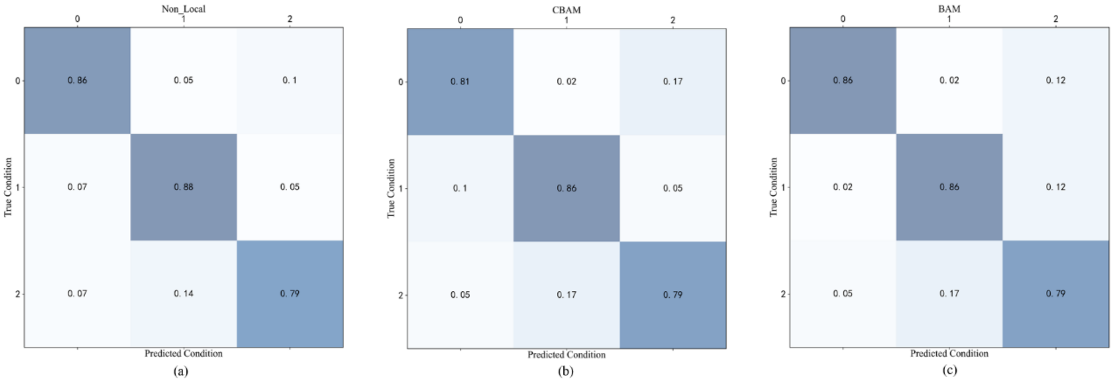

Fig. S6 (a), (b), (c) represent Non_Local, CBAM, BAM respectively

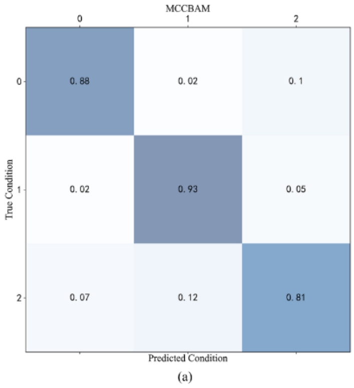

Fig. S7 (a) The attention mechanism proposed in this study, MCCBAM

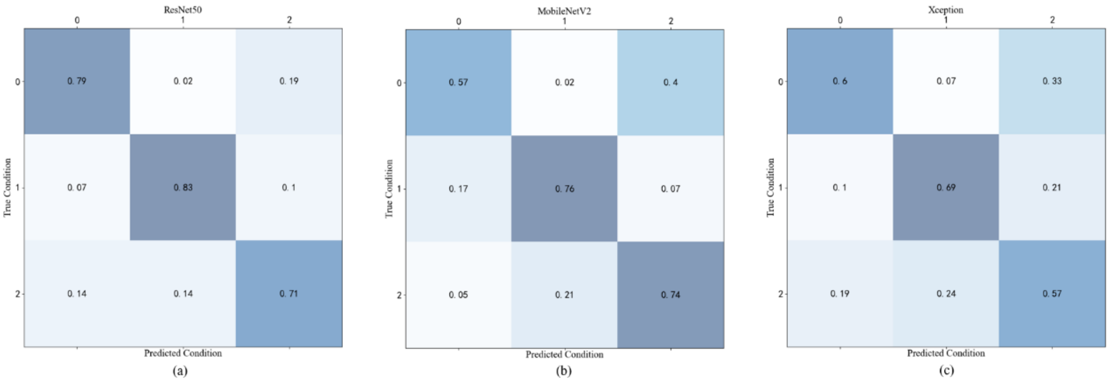

Fig. S8 (a), (b), (c) represent ResNet50, MobileNetV2, Xception respectively

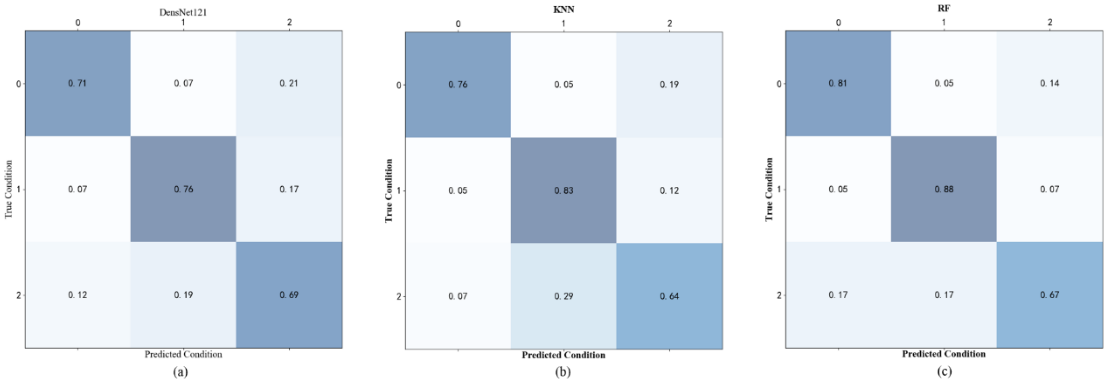

Fig. S9 (a), (b), (c) represent DensNet121, KNN, RF respectively

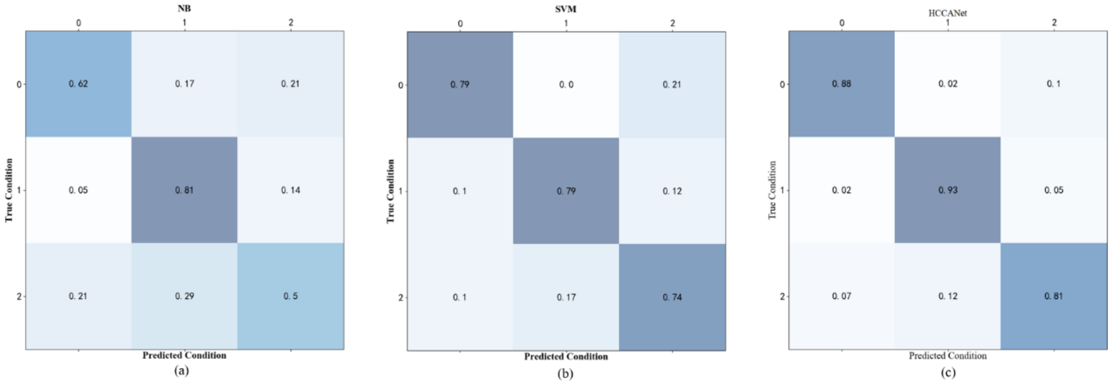

Fig. S10 (a), (b), (c) represent NB, SVM, HCCANet respectively
